# Impact of Exercise Modalities on Pentraxin-3 (PTX3) Levels: A Systematic Review and Meta-Analysis

**DOI:** 10.3390/muscles5010001

**Published:** 2025-12-23

**Authors:** Mohammad Rahman Rahimi, Hassan Faraji, Chenour Sadeghi, George John, Ildus I. Ahmetov, Hadi Golpasandi

**Affiliations:** 1 Department of Exercise Physiology, University of Kurdistan, Sanandaj 66177-15175, Iran; 2 Department of Physical Education and Sport Sciences, Mari.C., Islamic Azad University, Marivan 14778-93855, Iran; 3 Transform Specialist Medical Centre, Dubai 119190, United Arab Emirates; 4 Laboratory of Genetics of Aging and Longevity, Kazan State Medical University, 420012 Kazan, Russia; 5 Research Institute for Sport and Exercise Sciences, Liverpool John Moores University, Liverpool L3 5AF, UK

**Keywords:** Pentraxin-3, inflammation, exercise, athletes, endurance training, resistance training, obesity, overweight

## Abstract

Background: Pentraxin 3 (PTX3) is a key biomarker of innate immunity and inflammation, associated with muscle mass, metabolic syndrome, and obesity-related indicators. However, its role in training adaptations remains unclear, with studies reporting inconsistent PTX3 responses to acute and chronic exercise. This study aimed to compare the effects of aerobic exercise, resistance training, high-intensity interval training (HIIT), and acute exercise on PTX3 levels. Methods: A systematic search using Boolean logic was conducted in Web of Science, PubMed, and Google Scholar to identify randomized controlled trials examining the effects of exercise training and acute exercise on PTX3 levels. Results: Out of 3434 records published from 1992 to July 2025, 19 studies met the eligibility criteria. Meta-analysis revealed that aerobic training significantly increased PTX3 levels (SMD = 0.71; 95% CI, 0.173 to 1.252; *p* = 0.01; I^2^ = 83.14%), whereas resistance training significantly reduced them (SMD = −0.69; 95% CI, −1.025 to −0.370; *p* = 0.0001; I^2^ = 17.52%). HIIT did not elicit a significant change (SMD = 0.086; 95% CI, −0.364 to 0.535; *p* = 0.70; I^2^ = 0.00%). Notably, exercise training significantly elevated PTX3 in individuals over 50 years old (SMD = 1.124; 95% CI, 0.231 to 2.017; *p* = 0.014; I^2^ = 87.97%) but not in younger participants (SMD = −0.156; 95% CI, −0.640 to 0.327; *p* = 0.526; I^2^ = 78.80%). Conclusion: Aerobic and resistance exercise exert opposing effects on PTX3, suggesting distinct mechanisms through which different training modalities modulate inflammatory pathways relevant to muscle metabolism and repair. Acute exercise may also transiently elevate PTX3 to manage exercise-induced inflammation.

## 1. Introduction

Obesity, characterized by excessive fat accumulation, increases the risk of cardiovascular diseases, metabolic syndrome, diabetes, dyslipidemia, chronic kidney disease, hypertension, fatty liver, arthritis, asthma, and certain cancers, contributing to higher mortality rates. Epidemiological studies highlight a global rise in physical inactivity and obesity [1]. Excess macronutrient intake in adipose tissue triggers the release of pro-inflammatory cytokines, such as tumor necrosis factor-alpha (TNF-α) and interleukin-6 (IL-6), while reducing adiponectin production, promoting inflammation and oxidative stress [2].

Diabetes is a major public health concern, strongly associated with cardiovascular mortality. Approximately 80% of premature deaths and hospitalizations among individuals with diabetes result from coronary vascular complications. People with diabetes face a two- to fourfold higher risk of stroke, myocardial infarction, and cardiovascular mortality. Moreover, in those with both diabetes and coronary artery disease, conditions such as atherosclerosis, thrombosis, and inflammation are more severe [3]. Type 2 diabetes results from genetic predisposition and unhealthy lifestyle factors, involving multiple organs, including the pancreas, liver, skeletal muscles, kidneys, brain, small intestine, and adipose tissue. Elevated pro-inflammatory cytokines (e.g., TNF-α, IL-6) and reduced anti-inflammatory agents (e.g., interleukin-10, adiponectin) contribute to disease progression by impairing insulin production and increasing insulin resistance [4].

Cardiovascular disease remains the leading cause of death in individuals with type 2 diabetes, with obesity-related endothelial dysfunction playing a key role in both conditions. Inflammation is central to cardiovascular risk, driving atherosclerosis, coronary heart disease, thrombotic strokes, and cerebral aneurysms [5]. While acute inflammation restores tissue homeostasis by neutralizing harmful agents [2], chronic inflammation—marked by persistent immune activation and tissue remodeling—contributes to insulin resistance, diabetes progression, and atherosclerosis [6].

Pentraxin 3 (PTX3) is an emerging inflammatory biomarker and a member of the long pentraxin family. Unlike C-reactive protein (CRP), which is primarily produced in the liver as a systemic inflammatory response but can also be synthesized in extrahepatic tissues under certain conditions, PTX3 is rapidly synthesized at inflammation sites, reflecting localized tissue damage. PTX3 is expressed in various tissues and cells, including adipocytes, lungs, ovaries, thymus, brain, skeletal and cardiac muscle, visceral and subcutaneous fat, endothelial cells, fibroblasts, monocytes, macrophages, dendritic cells, and neutrophils [7,8]. PTX3 helps regulate inflammation in obesity by counteracting pro-inflammatory cytokines like IL-6 and TNF-α.

Importantly, PTX3 demonstrates a dual and context-dependent role. Although elevations in PTX3 have been linked to cardiovascular risk and adverse metabolic outcomes [9,10], PTX3 can also exert protective, anti-inflammatory, and vasculoprotective functions. These include modulating complement activation, promoting tissue repair, supporting endothelial function, and facilitating the resolution of inflammation [11]. Such protective actions are particularly relevant in physiological stress conditions, including aerobic exercise [12,13], high-intensity interval exercise [14] and total resistance exercises (TRX) [15]. Therefore, increases in PTX3 following physical activity may reflect adaptive, beneficial responses rather than worsening inflammation [16].

While CRP is a well-established cardiovascular risk marker, PTX3 may be a more reliable predictor of cardiovascular events [8]. PTX3 is implicated in atherosclerosis, acute coronary syndrome, and chronic heart failure [9,10], linking obesity, inflammation, and cardiovascular disease [17]. In healthy individuals, PTX3 levels are typically low but are associated with metabolic syndrome, insulin resistance, muscle mass, and obesity-related indicators, including BMI, waist-hip ratio, and visceral fat mass [18,19].

Exercise exerts anti-inflammatory effects, playing a crucial role in preventing and managing obesity, cardiovascular disease, diabetes, and related complications. Given PTX3’s involvement in inflammation and its context-dependent biological effects, its response to acute and long-term exercise has become an important research focus. Studies on PTX3 responses to exercise yield mixed results. One study reported a decrease in PTX3 levels after aerobic exercise at 75% maximal oxygen consumption in obese and normal-weight adults [20], while others observed increased PTX3 levels following submaximal aerobic exercise [13,21]. Long-term training studies, including 8-week [22], 10-week [23], and 12-week [24] high-intensity interval training (HIIT) interventions, found no significant changes in PTX3 levels. However, other research has reported both decreases [15,25] and increases [10,26] following regular exercise training.

Given these inconsistencies, this systematic review and meta-analysis aims to synthesize existing data on the effects of acute and long-term exercise on PTX3 levels, providing a clearer understanding of its role in exercise-induced inflammation and its potential dual actions in metabolic and cardiovascular health.

## 2. Materials and Methods

### 2.1. Protocol and Registration

This systematic review and meta-analysis was conducted following the Preferred Reporting Items for Systematic Reviews and Meta-Analyses (PRISMA) guidelines. The study protocol was registered with the University of York’s PROSPERO database (registration code: CRD42023472651). Since the analysis was based on aggregated data from previously published randomized controlled trials (RCTs), ethical approval was not required.

### 2.2. Including and Excluding Criteria

This systematic review and meta-analysis aimed to examine the effects of exercise training on circulating pentraxin 3 (PTX3) levels. Study selection followed the PICO (Population-Intervention-Comparator-Outcome) framework:

(1) Population: Healthy, overweight, and obese adults (≥18 years), including overweight individuals with diabetes, with any history of exercise training.
(2) Intervention: Any form of chronic exercise, regardless of duration or intensity.
(3) Comparator: Studies with a control group.
(4) Outcome: RCTs reporting PTX3 levels in both intervention and control groups.
(5) Study Design: Only RCTs were included.

Studies were excluded if they

(1) Were not RCTs.
(2) Included pediatric populations.
(3) Were conducted on animal models.

### 2.3. Study Search Strategy

A systematic search was performed using PUBMED, Google Scholar, and Web of Science, covering the period from 1992 to July 2025. The Boolean search method was applied using “AND,” “OR,” and “NOT” operators.

Keywords related to exercise training included “Exercise,” “Exercise training,” “Training,” “Physical activity,” “High-intensity interval training (HIIT),” “Resistance training,” “Weight training,” “Aerobic training,” and “Anaerobic training.” Keywords related to PTX3 included “Pentraxin 3” and “PTX3.” No language restrictions were applied to maximize the inclusion of relevant studies.

### 2.4. Study Selection Process

The study selection process followed these steps:

(1) Search results from all databases were imported into EndNote (version 20), and duplicate articles were removed.
(2) Two independent reviewers screened titles and abstracts to identify eligible studies. In cases of disagreement, a third reviewer resolved conflicts. Reasons for study exclusions were documented.
(3) Full texts of eligible studies were retrieved and reviewed according to the inclusion and exclusion criteria.
(4) The PRISMA 2020 flowchart illustrates the systematic selection process.

### 2.5. Data Extraction Process

Two independent authors extracted relevant data, including

(1) Study characteristics: Study type (single-group, two-group, random/non-random distribution), year of publication, first author.
(2) Participant characteristics: Age, BMI, health status.
(3) Exercise characteristics: Type, intensity, duration, frequency, and length of training programs.
(4) PTX3 measurements: Mean and standard deviation values at baseline and post-intervention for both exercise and control groups.

All eligible studies provided the necessary data for meta-analysis.

### 2.6. Quality Assessment

**Figure 1:**
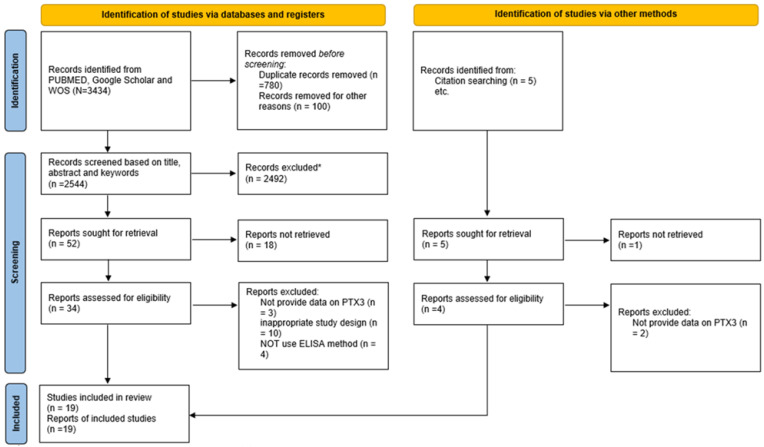
Flowchart illustrating the study selection process for investigating the effects of training on PTX3 levels.

Study quality was assessed using the PEDro checklist, with blinding-related items excluded due to limited feasibility in exercise interventions. Additionally, the Cochrane Risk of Bias 2 (RoB 2) tool [27] was used to evaluate bias in five domains (Figure 1):

(1) Randomization process.
(2) Deviation from intended intervention.
(3) Missing outcome data.
(4) Outcome measurement.
(5) Selective reporting.

Both reviewers independently assessed each study, remaining blinded to each other’s evaluations. Bias was classified as low risk, some concerns, or high risk.

### 2.7. Data Synthesis and Statistical Analysis

The extracted mean and standard deviation values were analyzed using CMA3 software V.4, which calculated the standardized mean difference (SMD) and 95% confidence intervals (CIs). Effect size interpretation followed Cochran’s guidelines, where an SMD of 0.2 was considered small, 0.5 medium, and 0.8 large. Heterogeneity among studies was assessed using the I^2^ test and Q-test. An I^2^ value below 50% indicated low heterogeneity, whereas a value above 50% suggested high heterogeneity, in which case a random-effects model was applied. Publication bias was evaluated using funnel plots and Egger’s test, with the trim-and-fill method used to correct potential bias. Subgroup analyses were conducted to examine variations based on exercise type (aerobic, resistance, or HIIT), exercise duration (less than eight weeks versus more than eight weeks), participant sex (male, female, or mixed), participant age (under or over 50 years), BMI categories, and population type (diabetic, obese, or overweight). All statistical analyses were performed using CMA3 software.

## 3. Results

### 3.1. Search Results and Study Selection

**Figure 2:**
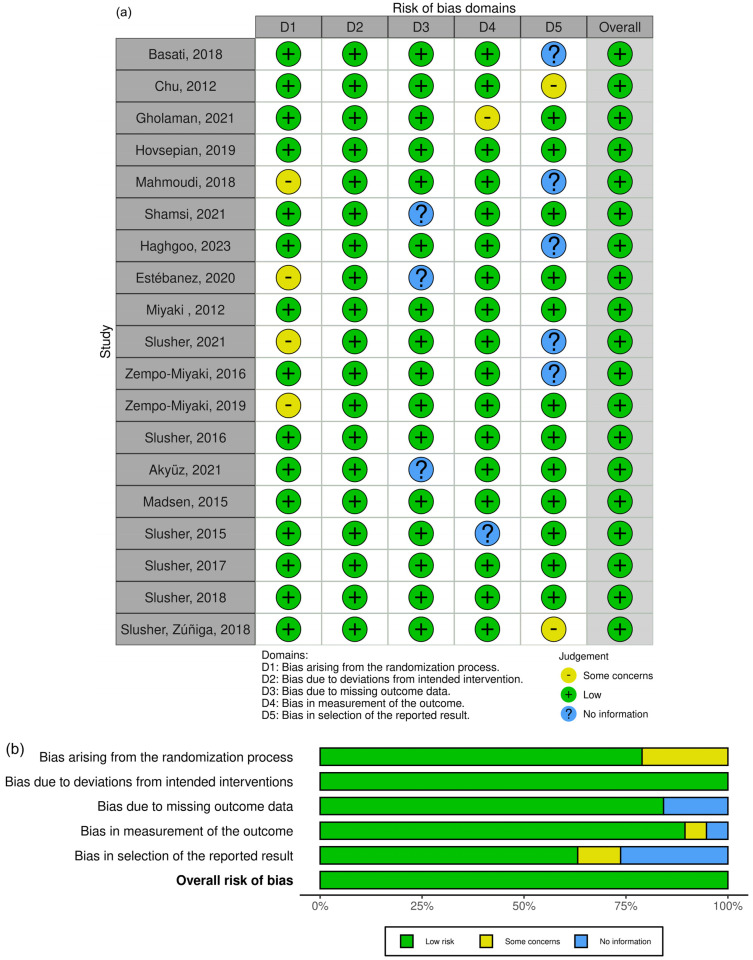
Traffic light plot of RoB 2 tool for risk of bias summary (**a**) and risk of bias graph (**b**).

**Figure 3:**
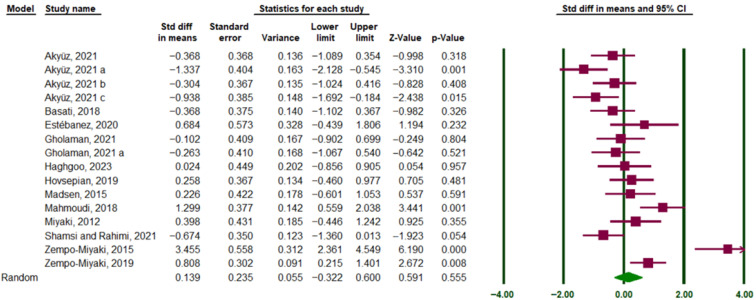
Forest plot representing 17 studies examining the impact of physical exercise on PTX3 protein levels in human blood circulation. All types of exercise (aerobic, resistance, and HIIT), with different durations of exercise (≤8 weeks and >8 weeks), were included. The green vertical lines denote the 95% confidence interval, while the solid brown squares represent the SMD. The green diamond illustrates the overall point estimate and 95% confidence interval derived from all included studies. Lowercase letters (a–c) indicate different subgroups reported within the same study. Arrows denote confidence intervals extending beyond the plotting range.

A comprehensive search of electronic databases, including PubMed, Google Scholar, and Web of Science, yielded a total of 3434 studies published between 1992 and July 2025. Following the removal of 890 duplicate studies, the remaining 2544 studies underwent an initial screening based on titles and abstracts. At this stage, 2492 studies were excluded due to ineligibility, leaving 52 studies for further evaluation. The full texts of these 52 studies were retrieved and thoroughly examined, resulting in the exclusion of 32 studies. Consequently, 19 studies remained for final inclusion (Figure 1). The risk-of-bias assessment performed using the RoB 2 tool is presented in the traffic light plot and summary graph, illustrating the overall judgment for each domain across the included studies (Figure 2). Supplementary Table S1 presents the methodological quality assessment of the studies using the PEDro scale. In the next stage, three studies were excluded due to a lack of necessary data [26,28,29], while two additional studies identified from other sources were deemed eligible. Ultimately, 19 studies met the inclusion criteria and were included in the analysis, all of which involved an exercise training group compared to a control group (Table 1). Notably, two of the selected studies included more than one training group (Figure 3). As of the end of July 2025, no additional articles meeting the inclusion criteria on the relevant topics had been published.

A random-effects model was applied.

### 3.2. Overall Effect of Long-Term Exercise Training on Circulating PTX3

This meta-analysis examined the effect of exercise training on circulating PTX3 protein levels, including 17 eligible studies. The overall effect size was calculated using the mean and standard deviation of PTX3 levels in both intervention and control groups, measured pre- and post-intervention. Standardized Difference in Means (SMD) was used to report the overall effect size. The heterogeneity analysis showed considerable variability among studies (I^2^ = 80.90%, Q-value = 83.74, df = 16, *p* = 0.0001). Therefore, a random-effects model was applied. The results demonstrated no significant effect of exercise training on PTX3 protein levels in circulation (SMD = 0.13, 95% CI: −0.256 to 0.516) (Figure 3).

**Figure 4:**
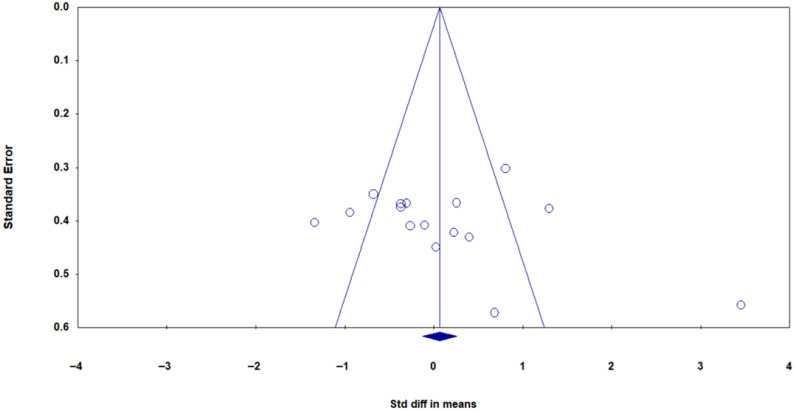
Funnel plot of meta-analysis findings on 17 studies examining the impact of physical exercise on PTX3 protein levels. Blue circles represent individual studies, and the blue diamond indicates the pooled effect estimate with its confidence interval. Visual analysis suggests symmetry and absence of publication bias.

Publication bias was assessed using a funnel plot (Figure 4) and Egger’s test. Visual analysis of the funnel plot suggested no asymmetry, and Egger’s regression test confirmed the absence of publication bias (B0 = 0.380, 95% CI: −2.63 to 3.41, t = 0.27, df = 15, *p* = 0.78).

### 3.3. Subgroup Analyses of Long-Term Exercise Training

#### 3.3.1. Effect of Exercise Type (Aerobic, Resistance, HIIT)

**Figure 5:**
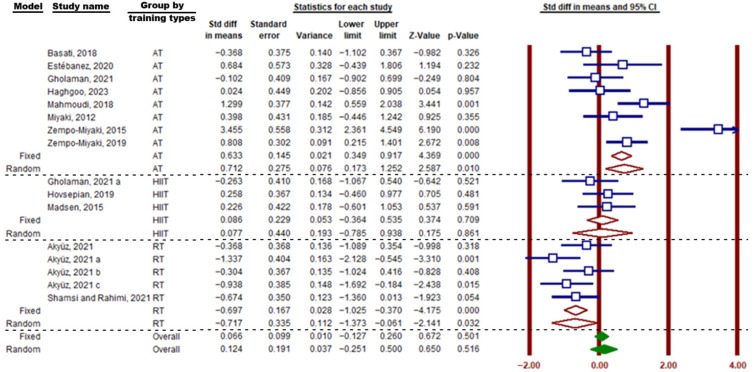
Forest plot related to the subgroup analysis of the impact of exercise type (AT: aerobic training, HIIT: high intensity interval training, RT: resistance training) on circulating PTX3 protein levels. Brown vertical lines represent the 95% confidence interval, while brown diamonds represent the standardized mean difference (SMD) from the random- and fixed-effects models of the subgroups. Solid green diamonds represent the overall point estimate and 95% confidence interval derived from all individual studies included in this meta-analysis. Lowercase letters (a–c) indicate different subgroups reported within the same study. Arrows denote confidence intervals extending beyond the plotting range.

In this meta-analysis, subgroup analyses were conducted based on the type of exercise (aerobic, resistance, and HIIT), duration of exercise (≤8 weeks and >8 weeks), gender of participants (both sexes, men, women), participants’ age (<50 years and ≥50 years), BMI, and participant type (diabetic, obese, and overweight). Regarding the subgroup analysis of the type of exercise, the findings indicate a significant increase in circulating PTX3 protein following aerobic training (SMD = 0.71; 95% CI, 0.173 to 1.252; *p* = 0.01; I^2^ = 83.14%) and resistance training (SMD = −0.69; 95% CI, −1.025 to −0.370; *p* = 0.0001; I^2^ = 17.52%). However, HIIT exercises (SMD = 0.086; 95% CI, −0.364 to 0.535; *p* = 0.70; I^2^ = 0.00%) had no effect on circulating PTX3 (Figure 5).

#### 3.3.2. Effect of Training Duration (≤8 Weeks vs. >8 Weeks)

**Figure 6:**
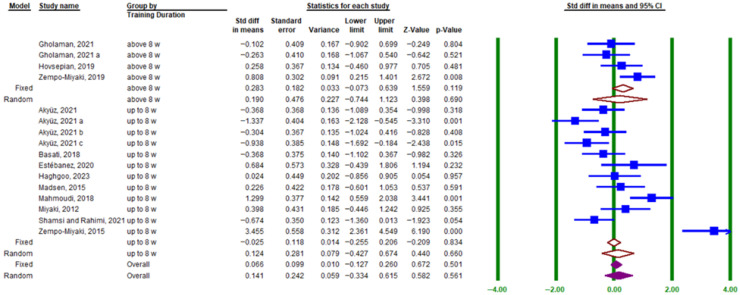
Forest plot related to the subgroup analysis of the impact of exercise duration on PTX3 protein levels in blood circulation. The green vertical lines represent the 95% confidence interval, and the solid blue squares indicate the standardized mean difference (SMD) of the individual studies. The hollow brown diamonds correspond to the SMD of the random- and fixed-effects model subgroups. Solid purple diamonds represent the overall point estimate and 95% confidence interval derived from all individual studies included in this meta-analysis. Lowercase letters (a–c) indicate different subgroups reported within the same study. Arrows denote confidence intervals extending beyond the plotting range.

The subgroup analysis related to exercise duration showed that training lasting ≤8 weeks (SMD = 0.124; 95% CI, −0.427 to 0.674; *p* = 0.669; I^2^ = 85.51%) and >8 weeks (SMD = 0.283; 95% CI, −0.073 to 0.639; *p* = 0.834; I^2^ = 47.17%) did not significantly affect circulating PTX3 protein levels (Figure 6).

#### 3.3.3. Effect of Participant Gender (Men, Women, Both)

**Figure 7:**
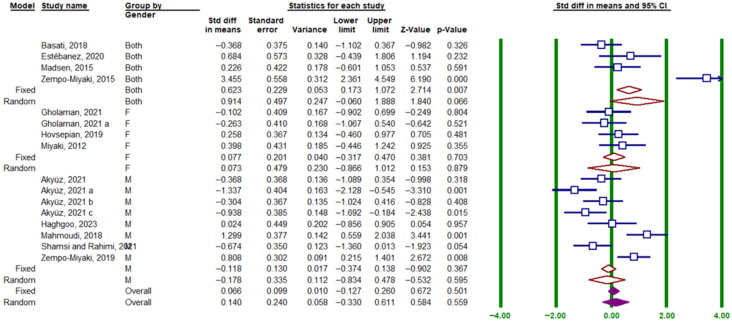
Forest plot related to the subgroup analysis of the impact of exercise on circulating PTX3 protein levels based on participant gender. Green vertical lines represent the 95% confidence interval, while brown diamonds indicate the SMD from the random- and fixed-effects model subgroups. Solid purple diamonds represent the overall point estimate and 95% confidence interval derived from all individual studies included in this meta-analysis. Lowercase letters (a–c) indicate different subgroups reported within the same study. Arrows denote confidence intervals extending beyond the plotting range.

The gender subgroup analysis indicated no significant difference in the effect of exercise on circulating PTX3 protein levels across studies: both sexes (SMD = 0.914; 95% CI, −0.060 to 1.888; *p* = 0.066; I^2^ = 91.08%), men (SMD = −0.178; 95% CI, −0.834 to 0.478; *p* = 0.595; I^2^ = 82.69%), and women (SMD = 0.077; 95% CI, −0.317 to 0.470; *p* = 0.641; I^2^ = 0.00%) (Figure 7).

#### 3.3.4. Effect of Participant Age (<50 Years vs. ≥50 Years)

**Figure 8:**
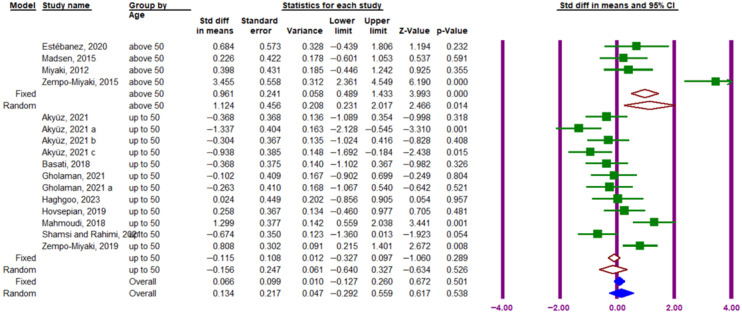
Forest plot related to the subgroup analysis of the impact of exercise on circulating PTX3 protein levels based on participant age. The purple vertical lines represent the 95% confidence interval, while the brown diamonds indicate the SMD from the random- and fixed-effects model subgroups. Solid blue diamonds represent the overall point estimate and 95% confidence interval derived from all individual studies included in this meta-analysis. Lowercase letters (a–c) indicate different subgroups reported within the same study. Arrows denote confidence intervals extending beyond the plotting range.

The subgroup analysis based on participant age showed a significant increase in circulating PTX3 protein in individuals aged ≥ 50 years (SMD = 1.124; 95% CI, 0.231 to 2.017; *p* = 0.014; I^2^ = 87.97%), but no significant effect in those aged < 50 years (SMD = −0.156; 95% CI, −0.640 to 0.327; *p* = 0.526; I^2^ = 78.80%) (Figure 8).

#### 3.3.5. Effect of BMI Categories

**Figure 9:**
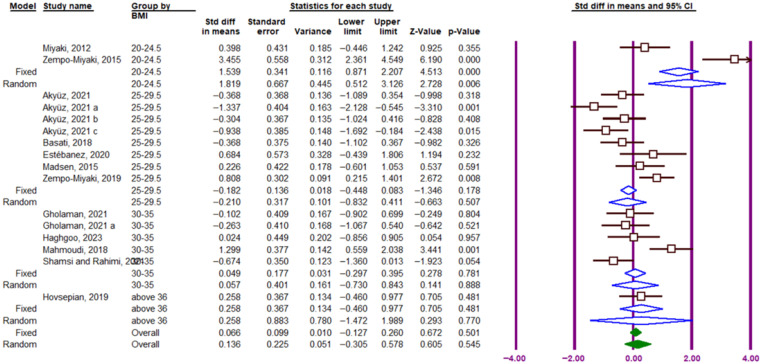
Forest plot related to the subgroup analysis of the impact of exercise on circulating PTX3 protein levels based on BMI. The purple vertical lines represent the 95% confidence interval, while the blue diamonds indicate the SMD from the random- and fixed-effects model subgroups. Solid green diamonds represent the overall point estimate and 95% confidence interval derived from all individual studies included in this meta-analysis. Lowercase letters (a–c) indicate different subgroups reported within the same study. Arrows denote confidence intervals extending beyond the plotting range.

Subgroup analysis by BMI indicated that PTX3 response to exercise was not affected by BMI. The effect of exercise in studies including participants with normal weight (BMI = 20–24.5; SMD = 1.819; 95% CI, 0.512 to 3.126; df = 1, *p* = 0.006; I^2^ = 94.68%), overweight (BMI = 25–29.5; SMD = −0.210; 95% CI, −0.832 to 0.411; df = 7, *p* = 0.507; I^2^ = 73.67%), first-degree obesity (BMI = 30–35; SMD = 0.057; 95% CI, −0.730 to 0.843; df = 4, *p* = 0.888; I^2^ = 74.89%), and BMI > 36 (SMD = 0.258; 95% CI, −0.460 to 0.977; df = 0, *p* = 0.481; I^2^ = 0.0001%) was not significant (Figure 9).

#### 3.3.6. Effect of Participant Health Status (Diabetic, Overweight/Obese)

**Figure 10:**
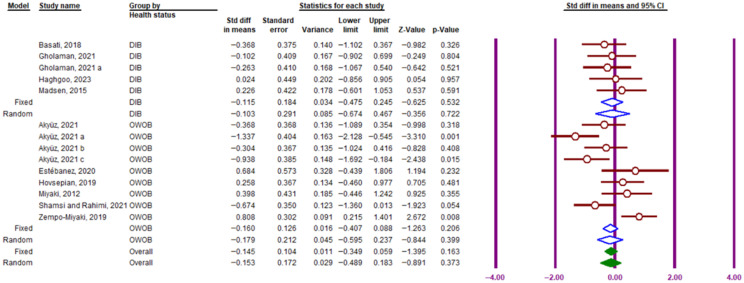
Forest plot illustrating the subgroup analysis of the impact of exercise training on circulating PTX3 protein levels based on participants’ health status (obese and overweight, diabetic). DIB, diabetes; OWOB, overweight and obesity. Lowercase letters (a–c) indicate different subgroups reported within the same study. The purple vertical lines represent the 95% confidence interval, while the blue diamonds indicate the standardized mean difference (SMD) from the random- and fixed-effects model subgroups. Solid green diamonds represent the overall point estimate and 95% confidence interval derived from all individual studies included in this meta-analysis.

A subgroup analysis based on health status included four studies of diabetic participants and six studies of overweight and obese participants. The findings showed that exercise significantly affected PTX3 blood circulation in people with diabetes (SMD = −0.115; 95% CI, −0.475 to 0.245; df = 4, *p* = 0.85; I^2^ = 0.0001%) and overweight/obese participants (SMD = −0.179; 95% CI, −0.595 to 0.237; df = 8, *p* = 0.399; I^2^ = 73.85%, Figure 10). However, three studies including healthy subjects, non-alcoholic fatty liver disease (NAFLD) patients, and postmenopausal women were excluded from the analysis.

### 3.4. Acute Effect of Exercise on Circulating PTX3

**Figure 11:**
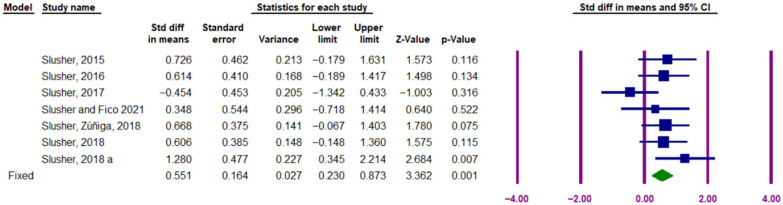
Forest plot illustrating the acute effect of exercise training on circulating PTX3 protein levels. The purple vertical lines represent the 95% confidence interval, while the solid squares indicate the standardized mean difference (SMD) from the fixed-effects model corresponding to individual studies. The solid green diamond represents the overall point estimate and 95% confidence interval derived from all individual studies included in this meta-analysis. Lowercase letter a indicate different subgroups reported within the same study.

The meta-analysis findings regarding the acute effect of exercise on PTX3 concentration in individuals are presented in Figure 8. The results of the I^2^ test indicated low heterogeneity among the studies included in the meta-analysis (I^2^ = 21.95%, Q-value = 7.688, df = 6, *p* = 0.262). Therefore, a fixed-effects model was applied to calculate the SMD effect size. The findings demonstrate a significant increase in circulating PTX3 protein levels in response to acute exercise, with an effect size of 0.551 (95% CI: 0.230 to 0.873, Z-value = 3.362, *p* = 0.001) (Figure 11).

**Figure 12:**
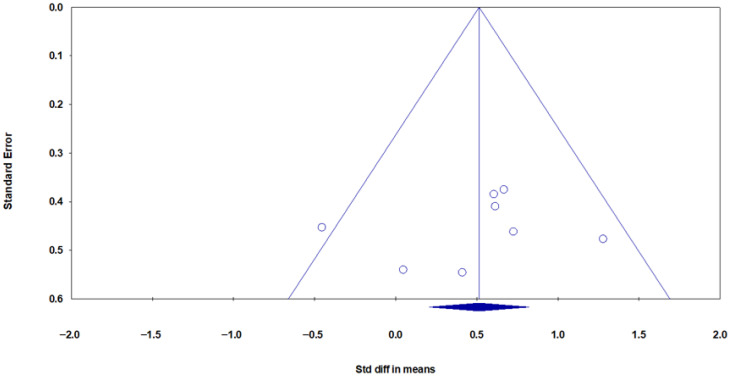
Funnel plot of meta-analysis findings related to the effect of acute exercise on circulating PTX3 protein levels. Blue circles represent individual studies, and the blue diamond indicates the pooled effect estimate with its confidence interval. Visual analysis of the plot indicates symmetry and the absence of publication bias among the studies.

Funnel plot visual analysis and Egger’s test indicated no publication bias among the studies (Figure 12). Egger’s regression test results confirmed the absence of publication bias, with a cut-off (B_0_) of −1.718 (95% CI: −9.36 to 5.92), t = 0.549, df = 6, *p* = 0.60.

## 4. Discussion

### 4.1. Key Findings

Evidence from studies spanning 1992 to 2025 can be summarized into five key findings. First, PTX3 response to exercise varies significantly: levels increase after endurance training, remain stable following HIIT, and decrease post-resistance training. Second, PTX3 increases more rapidly in individuals over 50 than in younger individuals. Third, gender and training duration (less than or more than eight weeks) do not influence PTX3 changes. Fourth, PTX3 alterations are independent of BMI, overweight, obesity, and diabetes status. Lastly, acute exercise is associated with elevated PTX3 levels.

### 4.2. Physiological Role of PTX3

PTX3, similar to CRP, is a key protein in innate immunity, tissue repair, and cancer-related processes. Its levels rise in response to inflammation, injury, atherosclerosis, and infections, whereas low PTX3 levels are linked to atherosclerosis progression, vascular inflammation, and macrophage accumulation [29,38]. PTX3 deficiency leads to increased expression of adhesion molecules, cytokines, and chemokines in the vascular wall, suggesting a potential role in regulating vascular inflammatory responses [38].

### 4.3. Dual and Context-Dependent Role of PTX3

Importantly, accumulating evidence indicates that PTX3 exhibits a dual and context-dependent role: elevated PTX3 levels in chronic cardiometabolic diseases reflect persistent inflammation, whereas exercise-induced increases likely represent an adaptive, protective mechanism supporting vascular homeostasis and immune regulation. This duality, conceptually similar to interleukin-6 behavior, underscores the importance of interpreting exercise-induced PTX3 elevations as a beneficial response rather than a pathological marker. Recognizing this bidirectional nature allows for better physiological and clinical interpretation of PTX3 modulation in response to physical activity.

### 4.4. Mechanisms Underlying Aerobic Exercise-Induced PTX3 Increases

This study found that both short- and long-term endurance training increase PTX3 levels. While the underlying mechanisms remain unclear, several potential pathways have been proposed. A recent review and meta-analysis indicated that sustained aerobic exercise enhances the expression and activity of eNOS—an enzyme responsible for nitric oxide production—as well as serum nitric oxide levels [39], potentially leading to increased PTX3 release [40]. Shear stress on human aortic endothelial cells activates nuclear factor κB and activator protein-1, triggering PTX3 gene expression [10]. Thus, chronic exercise-induced shear stress may contribute to elevated plasma PTX3 levels, reflecting a physiological anti-inflammatory and vasculoprotective adaptation. Clinically, this may translate into improved vascular resilience and lower cardiovascular risk in physically active individuals.

### 4.5. Vascular and Metabolic Implications

Cells within the vascular wall play a crucial role in integrating signals from various factors to regulate the inflammatory immune response. Through anti-inflammatory mechanisms, they fine-tune vascular inflammation, maintaining vascular integrity and homeostasis [41]. The increase in PTX3 levels following aerobic exercise may represent a protective physiological response against cardiovascular diseases, involving complement activation, opsonization, angiogenesis, and tissue repair. Additionally, endurance training-induced elevations in HDL-c [42] may stimulate PTX3 expression and release [41]. Thus, elevated PTX3 in the context of exercise should not be equated with systemic inflammation; instead, it may indicate improved vascular resilience and enhanced immunoregulatory capacity.

Research indicates a positive correlation between PTX3 and muscle GLUT4 protein, enhancing glucose uptake and transport to improve insulin sensitivity [43]. Elevated PTX3 levels post-aerobic exercise may also mitigate obesity-related inflammation in adipose tissue by modulating inflammatory pathways such as NF-κB and AMPK [44]. Moreover, the increase in PTX3 within adipose tissue of obese individuals via the neuropeptide Y (NPY) pathway may influence adipogenesis [45]. Exercise-induced rises in plasma PTX3 levels also correlate with myeloperoxidase, a marker of neutrophil activation. Neutrophils, a primary source of PTX3 post-exercise, may regulate their own infiltration as a feedback mechanism, reducing acute tissue damage caused by inflammation [29]. Persistent immune activation and tissue remodeling characteristic of chronic inflammation drive the development of insulin resistance, diabetes progression, atherosclerosis, and sarcopenia [6,46], highlighting the potential clinical significance of PTX3 modulation through exercise as a non-pharmacological strategy to improve metabolic and vascular health.

### 4.6. Resistance Training and PTX3 Modulation

Another key finding of this study was that PTX3 levels decreased following regular resistance training. This may result from the activation of central nervous system-mediated anti-inflammatory pathways, such as the vagus nerve and cholinergic reflex, which could inhibit PTX3 release [47]. Additionally, resistance training influences TLR2 receptor expression and function, modulating PTX3 and TNF-α levels through NF-κB signaling during inflammation triggered by TLR2 activation [48]. It also reduces TLR4 activity, potentially lowering PTX3 levels [49]. Notably, a meta-analysis revealed that acute aerobic exercise induces greater inflammation via TLR2 and TLR4 compared to resistance exercise, whereas chronic resistance training results in less inflammation than aerobic exercise [50]. These findings suggest that different exercise modalities can fine-tune PTX3-related inflammatory responses, which may have implications for tailoring exercise interventions in clinical populations to maximize anti-inflammatory benefits. Unlike CRP, which is reduced in response to both aerobic and resistance exercise [51], PTX3 shows opposing responses to these modalities, indicating that different types of training engage distinct inflammatory pathways relevant to muscle metabolism and tissue repair.

It should be noted that our analysis focused primarily on the type and duration of training and did not include stratification by other physiological indicators of adaptation, such as precise intensity measurements or combined circuit-training protocols. While some studies reported training intensities, inconsistencies in reporting prevented systematic inclusion of these data. Consequently, the present results should be interpreted as comparative effects of broad training modalities, rather than nuanced effects of intensity or mixed training regimens. Future studies providing detailed intensity metrics and separate analyses of circuit versus traditional resistance training would allow for more precise evaluation of PTX3 responses across exercise types.

### 4.7. HIIT and PTX3 Response

The findings indicated that HIIT had no statistically significant effect on circulating PTX3 levels (SMD = 0.086; 95% CI, −0.364 to 0.535; *p* = 0.70; I^2^ = 0.000%). While HIIT has demonstrated benefits in improving cardiometabolic parameters such as blood pressure, glucose levels, and body fat in overweight and obese individuals [52], its effects on inflammatory markers and PTX3 regulators—including C-reactive protein (CRP), interleukin-6 (IL-6), interleukin-10 (IL-10), and tumor necrosis factor alpha (TNF-α)—remain uncertain. Some studies suggest that prolonged HIIT reduces inflammatory markers in healthy or overweight/obese individuals [53], whereas others report no significant changes or even increases [23,52]. These discrepancies may arise from variations in HIIT intensity, duration, frequency, and type, as well as confounding factors such as diet, stress, and medication. The neutral effect of HIIT on PTX3 may indicate a balance between pro- and anti-inflammatory signaling, emphasizing the context-dependent nature of PTX3 modulation.

### 4.8. Age-Dependent PTX3 Modulation

An intriguing finding of this study was that exercise training significantly impacted individuals over 50 years of age, while no significant effect was observed in those under 50 (SMD = −0.156; 95% CI, −0.640 to 0.327; *p* = 0.526; I^2^ = 73.85%). Given that baseline PTX3 levels are higher in individuals over 50 and that oxidative stress and inflammation are more pronounced in older adults [22], it is plausible that aerobic exercise enhances PTX3 levels, potentially improving cardiovascular and metabolic health in this population. In older adults, the increase in PTX3 during exercise may counteract chronic low-grade inflammation (“inflammaging”), thereby reflecting a beneficial adaptive response rather than disease-associated elevation. However, further research is necessary to elucidate these effects.

### 4.9. Gender and Hormonal Influence on PTX3

Regarding gender differences, the findings suggest that exercise-induced PTX3 alterations are negligible. One possible explanation is that PTX3 is primarily synthesized by immune cells such as macrophages, dendritic cells, and neutrophils, rather than being directly influenced by sex hormones [54]. Consequently, PTX3 responses to exercise may depend more on immune system activation and regulation than on hormonal fluctuations. A study examining a 10-week HIIT program found that PTX3 levels decreased in sedentary overweight and obese women, regardless of menstrual cycle phase [48], suggesting that exercise had a more pronounced effect on PTX3 regulation than hormonal variations. Thus, compared to factors such as age, immune system function, and exercise characteristics, gender appears to play a minimal role in PTX3 modulation following chronic exercise. Nevertheless, further research is needed to confirm this hypothesis.

### 4.10. BMI, Obesity, and Metabolic Health

The findings indicate that changes in PTX3 levels were not significantly influenced by BMI, overweight and obesity, diabetes, or other diseases. Some studies suggest that obese individuals have lower PTX3 levels compared to those with normal weight [19], although this finding has not been consistently supported by other research [17]. However, the increase in PTX3 levels following acute aerobic exercise appears to be similar across individuals with normal weight, overweight, and obesity [13,21,29]. Therefore, both acute and chronic exercise seem to exert comparable effects on PTX3 levels, regardless of weight status. As with other subgroups, these responses likely reflect PTX3’s adaptive function during exercise, contrasting with its elevation during pathological inflammation.

Additionally, PTX3 levels tend to be higher in individuals with diabetes and lower in healthy individuals. Chronic exercise training has been shown to reduce PTX3 levels in individuals with diabetes while increasing PTX3 levels in healthy individuals, ultimately leading to similar PTX3 levels between both groups. Moreover, PTX3 has the ability to bind harmful molecules, such as advanced glycation end-products (AGEs), which are elevated in individuals with diabetes, and acts as an antioxidant and anti-inflammatory agent [22]. It also interacts with cytokines and receptors such as IL-6 and TLR4, which play key roles in regulating the inflammatory response to exercise, glucose metabolism, and insulin sensitivity [55]. This again illustrates PTX3’s context-specific behavior: elevated levels in diabetes may reflect pathological inflammation, whereas increases induced by exercise likely support metabolic regulation and tissue protection.

### 4.11. Acute Exercise-Induced PTX3 Increases

A meta-analysis of the data revealed that acute exercise resulted in a significant rise in PTX3 levels, with an effect size of 0.551 (95% CI: 0.230–0.873). This finding was supported by a Z-value of 3.362 and a *p*-value of 0.001. Numerous studies have examined exercise-induced increases in PTX3 and proposed various mechanisms underlying this effect. One proposed mechanism involves the release of PTX3 from peripheral neutrophils, which are activated by exercise-induced oxidative stress and cytokines. These activated neutrophils serve as a source of PTX3 in the bloodstream [29,56]. Additionally, PTX3 production by mononuclear cells, such as macrophages and dendritic cells, is influenced by aerobic fitness and exercise intensity [14]. Furthermore, activation of the PI3K/Akt signaling pathway in osteoblasts may contribute to increased PTX3 expression and enhanced bone mineralization [57]. The acute increase in PTX3 should therefore be interpreted as part of an adaptive immune response rather than a marker of harmful inflammation.

### 4.12. Methodological Quality of Included Studies

A critical consideration in interpreting these findings is the methodological quality of the included studies. Across the PEDro assessment, most trials demonstrated moderate methodological quality, with scores ranging from 6 to 8 out of 10, and none classified as low quality. Likewise, the RoB 2 analysis showed that the majority of studies were judged to have a low risk of bias across all key domains, with only a small number presenting “some concerns” or lacking information in areas such as outcome measurement or selection of reported results. Importantly, the direction and magnitude of PTX3 responses, particularly the increase following endurance training and the decrease after resistance training, were consistent across studies with low risk of bias, suggesting that the main findings are unlikely to be driven by methodologically weak trials. Conversely, studies with higher or unclear risk of bias did not disproportionately influence the pooled estimates, as their individual effects aligned with those from more rigorously conducted trials. Taken together, these quality assessments strengthen confidence in the overall conclusions, although minor uncertainties in a subset of studies indicate that future research with more robust blinding and allocation procedures would further refine the evidence base.

### 4.13. Limitations

Several limitations of this study should be acknowledged. First, substantial heterogeneity was present across the included studies due to differences in exercise protocols, intervention duration, training intensity, participant characteristics (e.g., age range, sex distribution, BMI, baseline fitness, and health status), and timing of PTX3 assessments. Such variability reduces the comparability of findings and limits the ability to draw uniform conclusions across training modalities. Second, many of the included trials had relatively small sample sizes, which reduces statistical power and increases the likelihood of type I and type II errors. The small cohorts also restrict the generalizability of the findings, and the results should not be extrapolated to the general population, particularly given that most participants were adults without severe chronic diseases and were often recruited from convenience samples. Third, there was variation in PTX3 measurement methods (e.g., different assay types, sensitivity thresholds, and sample processing protocols), which may introduce measurement bias and complicate cross-study comparisons. Fourth, a notable proportion of the available evidence originated from a limited number of research groups or geographic regions. This may introduce overrepresentation of specific methodological approaches and limit the external validity of the conclusions. Fifth, although the included studies generally demonstrated moderate to high methodological quality, residual confounding cannot be ruled out. Uncontrolled factors such as diet, circadian timing of blood sampling, medication use, baseline inflammatory status, and unreported training outside the interventions could influence PTX3 responses. Finally, because few studies directly compared multiple exercise modalities within the same population and long-term follow-ups were limited, causal inferences regarding chronic training effects remain tentative. Future well-powered, standardized, and multicenter trials are needed to confirm these findings and clarify the mechanisms through which different types of exercise modulate PTX3.

## 5. Conclusions

This study demonstrates that chronic endurance exercise training consistently increases circulating PTX3 levels, whereas regular resistance training results in their reduction. These changes likely reflect adaptive physiological responses, including anti-inflammatory and vasculoprotective effects, rather than pathological inflammation. Adaptations appear to be modulated by age and training duration, while gender exerts minimal influence. No significant associations were observed between PTX3 responses and health conditions such as diabetes, BMI, overweight, or obesity. High-intensity interval training (HIIT) did not significantly alter PTX3 concentrations. Importantly, a single bout of exercise reliably increased blood PTX3 levels, highlighting the acute sensitivity of this biomarker to physical activity. Clinically, these findings support the potential utility of PTX3 as a biomarker for monitoring exercise-induced immune and vascular adaptations and suggest that targeted exercise interventions could confer protective benefits against age- and disease-related inflammation.

## Figures and Tables

**Table 1:** Characteristics of the intervention studies included in the meta-analysis.

Study, Year, Country	Study Design	Subjects	Age (Mean: Years)	BMI (kg/m^2^)	Type of Exercise Training	Training Program Futures	Reduced PTX3
Basati, 2018 [30], Iran	RCT	59 CVD: Int G = 14 DIB/15 NDIB Co G = 15 DIB/15 NDIB	Int G = 58.65 Co G = 58.33	Int G = 26 ± 3.44 Co G = 26.1 ± 4.59	Aerobic Training	Total duration: 8 weeks Frequency a week: 3–4 sessions Duration per session: 60 min Intensity: 70% of maximum heart rate	Yes
Chu, 2012 [18], Korea	Single-group pre-post design	57 overweight or obese children (39 boys, 18 girls)	IntG = 12.04	Int G = 26.5	7 days of intense physical activity	Total duration: 1 week Frequency a week: 7 day Duration per session: NR Intensity: 1823 ± 134 kcal per day	Yes
Gholaman, 2021 [24], Iran	RCT	36 type 2 diabetic women MICT: 12 HIIT: 12 Co G: 112	Int G: MICT: 47.64 HIIT: 46.87 Co G = 46.35	Int G= Co G=	Continuous training with moderate-intensity (MICT) High intensity interval training (HIIT)	Total duration: 12-week Frequency a week: 3 Duration per session: MICT:47 min or running on treadmill with 60–70% of HRmax HIIT: 4 intervals (4 min) with 85–95% of maximum heart	Yes
Hovsepian, 2019 [23], Iran	RCT	30 overweight and obese female HIIT = 15 Co G = 15	Int G = 20.20 Co G = 20.7	Int G = 38.80 Co G = 38.90	High-intensity interval training (HIIT)	Total duration: 10 weeks Frequency a week: 4 Duration per session: 4 × 4 min Intensity: 90% max HR 3 × 3 min recovery by 70% max HR	No
Mahmoudi, 2018 [31], Iran	RCT	34 Non-alcoholic fatty liver disease (NAFLD) Int G = 17 Co G = 17	Int G = 41.5 Co G = 39.5	Int G = 30.16 Co G = 30.10	endurance training on a treadmill	Total duration: 8 weeks Frequency a week: 3 Duration per session: 45 min Intensity: 55–75%HRmax	No
Shamsi, 2021 [32], Iran	Single-group pre-post design	10 obese men	Int G = 31.87	Int G = 35.24	Circuit Resistance training	Total duration: 8 weeks Frequency a week: 3 Duration per session: 45 min Intensity: 65–80% 1RM	No
Haghgoo, 2023 [33], Iran	RCT	20 type 2 diabetic men Int G = 9 Co G = 11	Int G = 40.25 Co G = 40.25	Int G = 30.09 Co G = 30.09	Concurrent Training (Aerobic + Resistance taring)	Total duration: 6 weeks Frequency a week: 3 Duration per session: 25–35 min Intensity: Aerobic: 65–75% HRmax RT: 55–75 1RM	No
Estébanez, 2020 [22], Spain	RCT	elderly adults Int G = 9 Co G = 5	Int G = 68.67 Co G = 70.79	Int G = 25.71 Co G = 25.87	Aerobic exercise training cycling protocols on a stationary ergometer	Total duration: 8 weeks Frequency a week: 2 Duration per session: 25–30 min Intensity: 70–75% of maximum heart rate	Yes
Miyaki, 2012 [34], Japan	RCT	22 postmenopausal women Int G = 11 Co G = 11	Int G = 60 Co G = 60	Int G = 22.2 Co G = 22.4	aerobic exercise training	Total duration: 8 weeks Frequency a week: 3–5 Duration per session: cycling 30–45 min Intensity: 60–75 HRmax	No
Zempo-Miyaki, 2016 [10], Japan	RCT	32 healthy Japanese subjects Int G = 16 Co G = 16	Int G = 66.2 Co G = 66.2	Int G = 24.4 Co G = 21.1	aerobic exercise training	Total duration: 8 weeks Frequency a week: 3 days Duration per session: 45 min Intensity: 60–70% VO2peak	No
Zempo-Miyaki, 2019 [35], Japan	RCT	Forty-eight overweight and obese men Diet = 27 DietExe. = 21	Diet G = 48 Co G = 48	Diet G = 29.2 DietExe G = 29.2	the dietary modification (group D) or exercise training and dietary modification (group DE)	Total duration: 12 weeks Frequency a week: Duration per session: Intensity:	No
Slusher, 2016 [21], USA	RCT	25 untrained obese both sex: Int obese G = 13 (6 M/7F) normal-weight G = 12 (5 M/7 F)	Int obese G = 22.62 normal-weight G = 23.08	Int obese G = 35.35 normal-weight G = 21.86	an acute bout of maximal aerobic exercise (VO_2max_)	Total duration: Frequency a week: Duration per session: Intensity:	Yes
Akyüz, 2021 [25], Turkey	RCT	45 male participants: low-intensity resistance exercise group (LIEG): 15 moderate-intensity resistance exercise group (MIEG): 15 control group (CG): 15	mean 41 years	LIEG = 27.7 MIEG = 27.8 CG = 25.7	Resistance training	Total duration: 8 weeks Frequency a week: 3 Duration per session: lasted about 60 min Intensity: MIEG: 8–10 repeats at 70–80% 1RM LIEG: 15–17 repeats at 50–60% 1RM.	Yes
Madsen, 2015 [36], Denmark	Two-groups pre-post design	T2D: 10 CON: 13 matched	Int G = 56 Co G = 52	Int G= Co G=	low volume high intensity interval training (HIIT) r (3 weekly sessions: 10 60 s HIIT) on a cycle ergometer	Total duration: 8 weeks Frequency a week: 3 Duration per session: 10 × 60 s Intensity: 1 min of active recovery	No
Slusher, 2015 [13], USA	Two-groups pre-post design	Obese: 10 [4 M/6F] normal-weight: 10 [4 M/6F]	Obese G = 21.5 normal-weight = 23.2	Obese G = 35.04 normal-weight G = 21.87	Acute continuous submaximal aerobic exercise	Total duration: Frequency a week: 1 session Duration per session: 30 min Intensity: 75% VO2max	No
Slusher, 2017 [20], USA	Two-groups pre-post design	Obese: 10 normal-weight: 10	Obese G = 22.8 normal-weight = 23.27	Obese G = 36.08 normal-weight G = 21.89	acute bout of aerobic exercise at	Total duration: Frequency a week: 1 session Duration per session: 12 to 15 min Intensity: 75% VO2max	Yes
Slusher, 2018 [14], USA	Crossover design	Eight healthy male subjects	24.5	25.18	Acute high-intensity interval exercise (HIIE) and continuous moderate intensity exercise (CMIE)	Total duration: 1 session Frequency a week: 1 Duration per session: Intensity: HIIE—10 high-intensity intervals of cycling for 60 s at 90% Wmax separated by 2 min of active rest CMIE—28 min of continuous exercise at 60% Wmax.	No
Slusher, Zúñiga, 2018 [37], USA	Two-groups pre-post design	aerobically trained (AT) = 15 untrained (UT) = 15	AT G = 25.27 UT G = 25.27	AT G = 21.68 UT G = 22.18	Graded treadmill exercise test	Total duration: Frequency a week: Duration per session: Intensity:	No

Abbreviations: DIB: Diabetics; Int G: Intervention Group; Co G: Control Group; CVD: Cardiovascular Disease; RCT: Randomized Controlled Trial; NR: Not Reported; NDIB: Non-Diabetics; BMI: Body Mass Index; HRmax: Maximum Heart Rate; VO_2_peak: Peak Oxygen Uptake; 1RM: One-Repetition Maximum; MICT: Moderate-Intensity Continuous Training; HIIT: High-Intensity Interval Training; HIIE: High-Intensity Interval Exercise; CMIE: Continuous Moderate-Intensity Exercise; RT: Resistance Training; T2D: Type 2 Diabetes; NAFLD: Non-Alcoholic Fatty Liver Disease; AT: Aerobically Trained; UT: Untrained; CON: Control; Wmax: Maximum Workload; LIEG: Low-Intensity Exercise Group; MIEG: Moderate-Intensity Exercise Group; CG: Control Group; Diet G: Diet-only Group; DietExe G: Diet and Exercise Group.

## Data Availability

As this is a meta-analysis, all data included in this study are available in the cited references.

## Supplementary Materials

The following supporting information can be downloaded at: https://www.mdpi.com/article/10.3390/muscles5010001/s1, Table S1: Methodological quality assessment of the studies using the PEDro scale.

## Abbreviations

The following abbreviations are used in this manuscript:

AGEs	Advanced Glycation End-products
BMI	Body Mass Index
CI	Confidence Interval
CMIE	Continuous Moderate-Intensity Exercise
CRP	C-Reactive Protein
CVD	Cardiovascular Disease
GLUT4	Glucose Transporter Type 4
HDL-c	High-Density Lipoprotein Cholesterol
HIIT	High-Intensity Interval Training
HIIE	High-Intensity Interval Exercise
HRmax	Maximum Heart Rate
IL-6	Interleukin-6
IL-10	Interleukin-10
MICT	Moderate-Intensity Continuous Training
NAFLD	Non-Alcoholic Fatty Liver Disease
NF-κB	Nuclear Factor Kappa-Light-Chain-Enhancer of Activated B Cells
NPY	Neuropeptide Y
PI3K/Akt	Phosphoinositide 3-Kinase/Protein Kinase B
PTX3	Pentraxin-3
RCT	Randomized Controlled Trial
RM	Repetition Maximum
T2D	Type 2 Diabetes
TLR2/4	Toll-Like Receptor 2/4
TNF-α	Tumor Necrosis Factor-Alpha
VO_2_max	Maximal Oxygen Uptake
Wmax	Maximum Workload
