# Supplementary material for: Impact of Exercise Modalities on Pentraxin-3 (PTX3) Levels: A Systematic Review and Meta-Analysis
Source: Muscles. 2025 Dec 23;5(1):1. doi: 10.3390/muscles5010001 (PMC12821439; doi:10.3390/muscles5010001)
Supplement: Supplementary file 1 [file muscles-05-00001-s001.zip › muscles-3911120-supplementary.pdf]

Supplementary materials

Supplementary Table S1. Methodological quality assessment of the studies using the PEDro scale

Study	Eligibility criteria	Random Allocation	Concealed allocation	Baseline comparability	Blind subjects	Blind assessors	Outcome measures assessed in 85% of participant	Intention-to-treat analysis	Between-group comparisons	Point estimates and variability	Total score
Basati, 2018	+	−	−	+	−	−	+	+	+	+	6
Chu, 2012	+	−	+	+	−	−	+	+	+	+	7
Gholaman, 2021	+	+	−	+	−	−	+	+	+	+	7
Hovsepian, 2019	+	+	−	+	−	−	+	+	+	+	7
Mahmoudi, 2018	+	+	−	+	−	−	+	+	+	+	7
Shamsi, 2021	+	+	−	+	−	+	+	+	+	+	8
Haghgoo, 2023	+	+	−	+	−	−	+	+	+	+	7
Estébanez, 2020	+	−	−	+	−	−	+	+	+	+	6
Miyaki , 2012	+	+	−	+	−	−	+	+	+	+	7
Slusher, 2021	+	−	−	+	−	−	+	+	+	+	6
Zempo-Miyaki, 2016	+	+	−	+	−	−	+	+	+	+	7
Zempo-Miyaki, 2019	+	−	−	+	−	−	+	+	+	+	6
Slusher, 2016	+	−	−	+	−	+	+	+	+	+	7
Akyüz, 2021	+	+	−	+	−	−	+	+	+	+	7
Madsen, 2015	+	−	−	+	−	−	+	+	+	+	6
Slusher, 2015	+	+	−	+	−	−	+	+	+	+	7
Slusher, 2017	+	−	−	+	+	−	+	+	+	+	7
Slusher, 2018	+	−	−	+	−	−	+	+	+	+	6
Slusher, Zúñiga, 2018	+	−	−	+	−	−	+	+	+	+	6
